# A New Paradigm of Magnetron Target Design

**DOI:** 10.3390/nano16090543

**Published:** 2026-04-29

**Authors:** Viktor I. Shapovalov, Daniil S. Sharkovskii, Joshua K. Zephaniah, Arseniy V. Nikolaev

**Affiliations:** Faculty of Electronics, St. Petersburg Electrotechnical University “LETI”, Prof. Popov Str., 5F, St. Petersburg 197022, Russia; sharkovskiy.d@yandex.ru (D.S.S.); zephaniahk333@gmail.com (J.K.Z.); nikolaev.a2004@mail.ru (A.V.N.)

**Keywords:** equiatomic alloy, film, magnetron sputtering, multilayer target, titanium, tantalum, niobium, molybdenum

## Abstract

This communication discusses the problem of depositing equiatomic metal alloy films. It is shown that this problem can be solved using a magnetron equipped with a target constructed using a new “multilayer target” paradigm. This target, sputtered in an argon environment, consists of several parallel metal plates mounted on the magnetron axis. A method based on the equality of the sputtered fluxes generated by the plates is proposed for calculating the geometric dimensions of the plates. This equality leads to a system of algebraic equations, which are proposed to be solved under the assumption of a uniform discharge current density distribution in the sputtering region of the target. The communication describes two types of targets in which the plates have slots of different shapes. In one case, the slots are shaped as sectors of a ring with a given angle. In the other, the plates are shaped as rings. As examples, the geometric dimensions of targets for a balanced magnetron system intended for the deposition of films of equiatomic Ti_0.33_Ta_0.33_Nb_0.33_ and Ti_0.25_Ta_0.25_Nb_0.25_Mo_0.25_ alloys are calculated. The presentation is accompanied by the results of individual experiments. This report is preliminary in nature; experimental verification is ongoing. The application of the new paradigm in magnetron target design facilitates the fabrication of films of nanostructured medium- and high-entropy alloys with specified chemical compositions, which is the central theme of the Special Issue devoted to functional nanomaterials.

## 1. Introduction

### 1.1. Equiatomic Alloy Films

**Figure 1:**
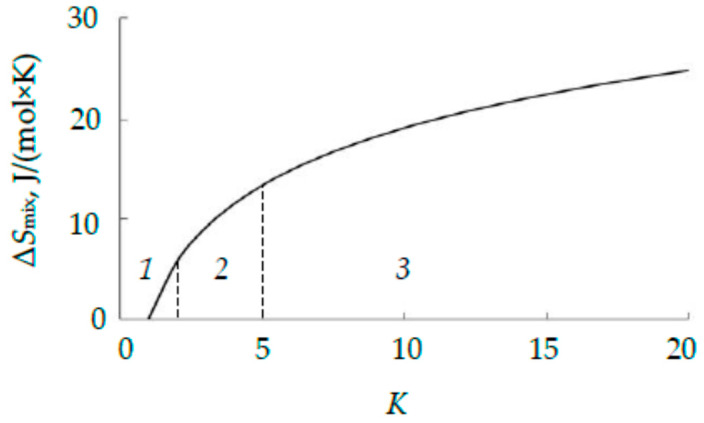
Dependence of the Δ*S*_mix_ value on the number of components of an equiatomic alloy. Regions: *1*—low-; *2*—medium-; *3*—high-entropy alloys.

About several millennia, humans have traditionally tailored metallic properties by microalloying a single base metal (e.g., Cu, Fe, Al). Approximately two decades ago, however, a new approach for alloy design emerged [1]. It was based on mixing several metals in equiatomic or near-equiatomic ratios [2]. A defining characteristic of these alloys is their high entropy of mixing Δ*S*_mix_ [3]:

(1) $$\Delta S_{\mathrm{mix}}=R\ln K\;,$$

where *R* = 8.314 J/(mol × *K*) is the universal gas constant; *K* is the number of components in the alloy. The value (1) increases, as shown in Figure 1. Alloys with *K* ≥ 5 (Δ*S*_mix_ ≥ 1.61*R*) are usually called high-entropy; those with 2 ≤ *K* < 5 (0.69*R* ≤ Δ*S*_mix_ < 1.61*R*) are called medium-entropy; and, finally, traditional and two-component alloys are called low-entropy [4].

The deposition of equiatomic alloy films has been established as an effective method for enhancing the functional and mechanical properties of various substrate materials [4].

The development of alloy-film materials science is exemplified by titanium (Ti) [5]. Interest in Ti has progressed from studies of pure-metal films [6], through investigations of simple chemical compounds (e.g., oxides or nitrides [7,8,9,10,11]), to work on complex solid solutions of binary (e.g., TiAlN [12], TiCrN [13]) and ternary (e.g., NiTiCrN, MoTiCrN [14]) systems. One effective development strategy involves the Ti-Ta binary system [15,16,17]. Further improvements have been demonstrated through the strategic addition of a third component to the Ti-Ta system [18,19,20].

Further advances in materials science have naturally broadened the equiatomic-alloy concept to encompass high-entropy systems [21,22,23].

### 1.2. Film Deposition

Magnetron sputtering of metal targets in an argon environment is often used to deposit multicomponent alloy films. Typically, either a pre-doped [24] or a sectioned target [25] is used. Co-sputtering, which involves multiple magnetrons with targets of different compositions, is more complex [26].

When using these methods, certain difficulties arise. Fabricating an alloy target involves complex vacuum melting technologies. In addition to the process difficulties and equipment complexity, a significant drawback is the lack of flexibility in composition selection, since any change in film composition requires melting a new target. Furthermore, alloy sputtering can continuously deplete the target surface of the component with the highest sputtering yield. In such cases, the target and sputtering flux compositions are not identical, leading to changes in film composition. Sectional (or mosaic) targets present an alternative solution. Their fabrication requires solving the complex engineering problem of reliably mounting various single-element components on a cooled plate while achieving high thermal contact and ensuring mechanical stability under significant thermal loads.

The complexity of a co-magnetron sputtering system containing multiple magnetrons is obvious. First, it must be equipped with an appropriate number of high-voltage power supplies. Secondly, it requires complex design solutions to ensure uniformity and homogeneity of the films.

In recent years, an alternative approach has been proposed using a magnetron equipped with a target constructed based on a new “multilayer target” paradigm [27,28,29,30,31]. Such a target consists of several parallel metal plates mounted on the magnetron axis. Various designs have been investigated, the simplest of which is a two-layer configuration. In this configuration, the inner plate, cooled by running water, acts as a refrigerator. The outer plate operates in hot mode and generates a flux of the working substance that forms the film. To deposit the alloy film, the target is equipped with an appropriate number of metal plates fixed on a single axis. Moreover, each of them (except the inner one) has holes. Through these, the sputtering is released onto the underlying surface.

This communication demonstrates that the problem of equiatomic alloy film deposition can be solved using a magnetron equipped with a target designed using the new “multilayer target” paradigm. Two types of targets are described. In one case, the slots in the outer plates are shaped as ring sectors with a given angle. In the other case, the central region is removed from the plates, resulting in a ring-shaped plate. As an example, we calculate the geometric dimensions of targets for a balanced magnetron system designed to deposit Ti_0.33_Ta_0.33_Nb_0.33_ and Ti_0.25_Ta_0.25_Nb_0.25_Mo_0.25_ equiatomic alloy films. This communication describing targets with slots of a different shape is preliminary in nature. Experimental verification is ongoing. The application of the new paradigm in the design of magnetron targets facilitates the fabrication of films of nanostructured medium- and high-entropy alloys with a given chemical composition, which is the central theme of the Special Issue devoted to functional nanomaterials.

## 2. Three-Layer Target

### 2.1. Slot Shapes

**Figure 2:**
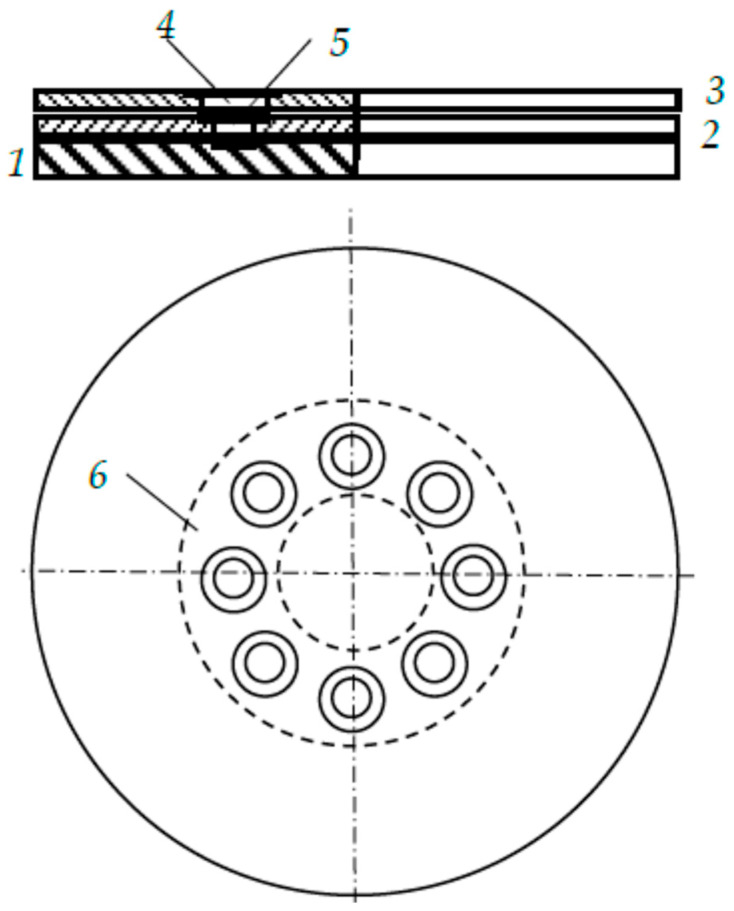
Schematic representation of a three-layer magnetron target: *1*–*3*—plates made of different metals; *4* and *5*—slots; *6*—sputtering area of the target.

**Figure 3:**
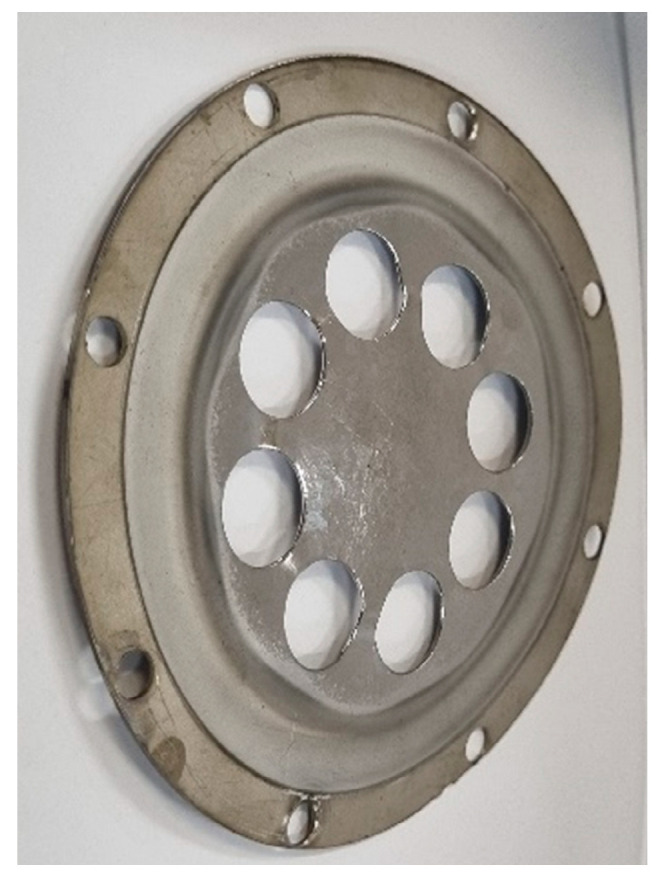
Photograph of the outer 2 mm thick plate of a three-layer target containing slots, the total area of which occupies half of the sputtered area.

In the first versions of multilayer targets, the slots in the plates were made in the form of holes located symmetrically relative to the magnetron axis [28]. A schematic representation of a three-layer target designed for deposition of a three-component alloy is shown in Figure 2. A photograph of the outer plate with the maximum possible area of circular slots is shown in Figure 3.

Clearly, to deposit a film of an equiatomic alloy containing transition metals with similar sputtering yields, the sputtering zones of each target plate must have approximately equal areas. Figure 2 and Figure 3 show that a three-layer target with round slots is unsuitable for this task. This communication proposes targets with slots of a different shape.

**Figure 4:**
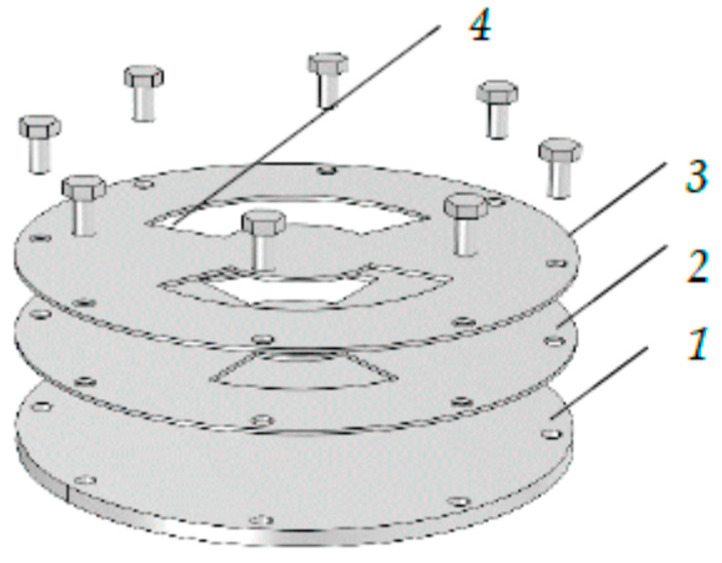
Schematic representation of a three-layer target: *1*, *2* and *3*—inner, middle and outer plates, respectively; *4*—slot.

A schematic representation of a target designed for depositing a film of the M_1_M_2_M_3_ alloy is shown in Figure 4.

The target contains three metal sputtering plates *1*–*3* on one axis, attached to the magnetron housing. The annular area of the magnetron target sputtered by argon ions is bounded by the inner and outer radii *R*_01_ and *R*_02_, respectively. It includes the sputtered areas of all wafers, which we will henceforth refer to as zones. This area includes the sputtering zones of all plates. The inner plate *1* made of metal M_1_ is cooled by running water. The middle plate *2* made of metal M_2_ and the outer plate *3* made of metal M_3_ contain two slots *4* each, made in the sputtering zone. The slots in each plate are located symmetrically relative to the center of the target. They have the shape of ring sectors with a given angle. We will designate them as α_2_ for the middle plate and as α_3_ for the outer plate, and will henceforth specify them in degrees. The specified angles define the sputtering zones on each plate *s_i_*, *i* = 1, 2, 3. The specified values of the subscripts for *s_i_* correspond to the plate numbers in Figure 4.

**Figure 5:**
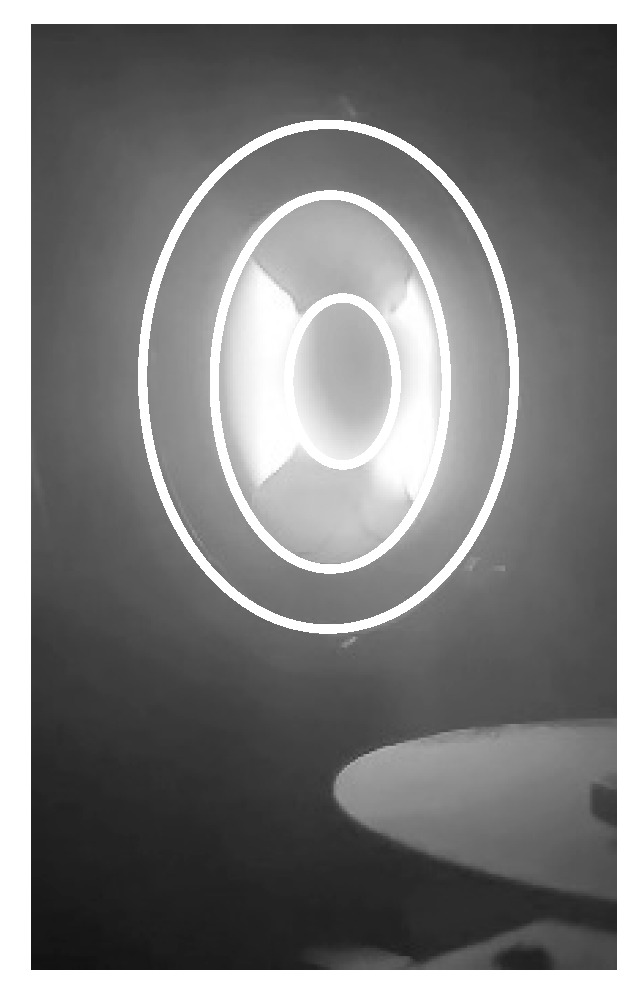
Photo of a working magnetron with a three-layer target.

**Figure 6:**
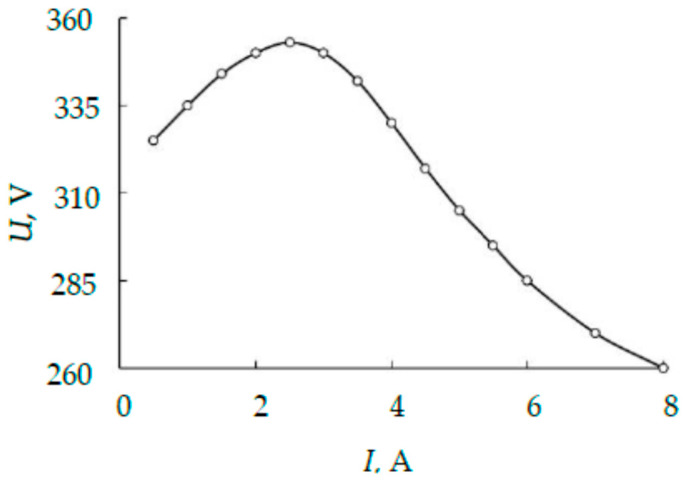
Current-voltage characteristic of a magnetron with a three-layer target (dots—experiment, solid line—trend).

Figure 5 shows a photograph of an operating magnetron with a three-layer target, which is described in Section 2.3. TiTaNb alloy film. The inner ring in Figure 5 reflects the state of the heated outer plate. Dark areas in this ring correspond to slots in the plate. Light areas reflect the heating of the plate. The significant temperature of the outer plate is indicated by the current-voltage characteristic shown in Figure 6. The dependence in Figure 6 was obtained by studying the discharge of a magnetron operating on direct current in an argon environment at an operating pressure of 5 mTorr. The direct current source feeding the magnetron made it possible to perform measurements by varying the discharge current from 1 to 8 A in 0.5 A increments (shown by dots in Figure 6).

The maximum in Figure 6 in the discharge current region of approximately 3 A indicates an increase in thermionic emission of the outer plate to a significant level, where it becomes similar to ion-electron emission [32].

Initial experiments with multilayer targets (see Figure 2 and Figure 4) revealed that:

(1) The magnetron discharge with such targets is stable;

(2) Although the current–voltage curve (Figure 6, I > 3 A) exhibits a segment typical of arc discharge, no droplet phase was observed on the quartz-glass and polished-silicon substrates.

Furthermore, in experiments with an outer plate up to 1.5 mm thick, significant deformation of its central region was observed. As the plate thickness increased to 3 mm, the deformation disappeared. Electrical contact between the plates was not disrupted, as it was maintained by mounting bolts located on the periphery of the target.

### 2.2. Three-Layer Target Model

**Figure 7:**
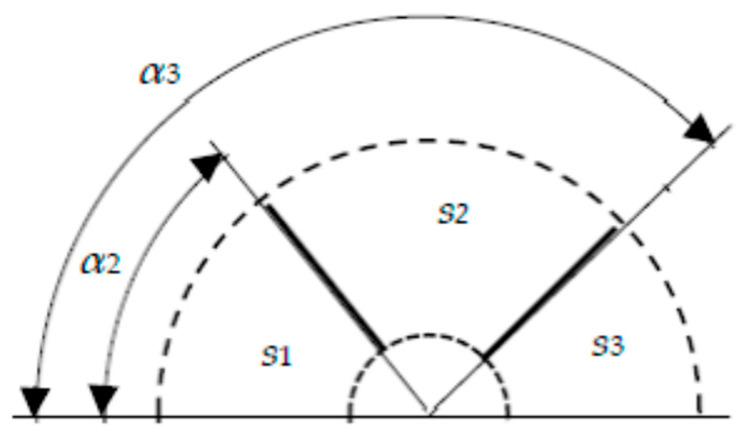
Schematic representation of half the sputtering area of a three-layer target: *s_i_*, *i* = 1, 2, 3—sputtering zone of the inner, middle and outer plates, respectively; α_2_ and α_3_—angles of the sector slots in the middle and outer plates, respectively.

To analyze the flux distribution produced by the three-layer target, Figure 7 depicts one half of the annular sputtering region, bounded by dashed lines at characteristic radii *R*_01_ and *R*_02_.

This representation is quite sufficient, since the target has axial symmetry. The slot in the middle plate has an angle of α_2_, and in the outer one—α_3_. In Figure 7, the symbols *s_i_ i* = 1, 2, 3 indicate the sputtering zones regions of all plates. The value of the index *i* corresponds to the plate: 1—inner; 2—middle; 3—outer.

We further use the new designations α2 and sα3—the areas of sectors with angles α_2_ and α_3_, cut out in the middle and outer plates, respectively. It is well known that the area of a sector with an angle α given in degrees, cut in a circle with radius *R*, is equal to $\pi R^{2}\alpha /360$. Using this formula, we write an expression for calculating the area of the slot on the *i*-th plate (see Figure 7):

(2) $$s_{\alpha i}=\frac{\pi \alpha_{i}}{180}\left(R_{02}^{2}-R_{01}^{2}\right),\;i=2,\;3.$$

Next, using (2), we establish a relationship between the target parameters. Figure 7 makes it easy to determine the areas of the sputtering regions on the plates:

(3) $$\begin{array}{l} s_{1}=s_{\alpha 2}=\frac{\pi \alpha_{2}}{180}\left(R_{02}^{2}-R_{01}^{2}\right)=\frac{\alpha_{2}s}{180},\\ s_{2}=s_{\alpha 3}-s_{\alpha 2}=\frac{\pi }{180}\left(R_{02}^{2}-R_{01}^{2}\right)\left(\alpha_{3}-\alpha_{2}\right)=\frac{\left(\alpha_{3}-\alpha_{2}\right)s}{180},\\ s_{3}=s-s_{\alpha 3}=s\left(1-\frac{\alpha_{3}}{180}\right). \end{array}$$

In (3), the total area of the target’s sputtering area is denoted by $s=\pi \left(R_{02}^{2}-R_{01}^{2}\right)$. Each plate, due to the effect of the argon ion flux, becomes a metal source with a flux density $J_{i}\left(r,\;\varphi ,\;z\right),\;i=1,\;2,\;3,$ which we define in a cylindrical coordinate system:

(4) $$J_{i}\left(r,\;\varphi ,\;z\right)=\frac{S_{i}j\left(r,\;\varphi \right)}{e(1+\gamma_{i})}=a_{i}\frac{j\left(r,\;\varphi \right)}{e},\;i=1,\;2,\;3,$$

where *r* is the distance from the origin to a given point in the vacuum chamber in cm; φ is the polar angle; *z* is the projection of the given point onto the normal to the target surface; *S_i_* and *γ_i_* are the sputtering yield and ion-electron emission coefficients of the *i*-th metal; $j\left(r,\;\varphi \right)$ is the distribution of discharge current density in the annular sputtering zone (given in polar coordinates at *z* = 0); *e* = 1.6 × 10^−19^ C is the electron charge. In (4), the notation ai is used, *i* = 1, 2, 3:

(5) $$a_{i}=\frac{S_{i}}{\left(1+\gamma_{i}\right)},\;i=1,\;2,\;3.$$

By establishing that a flux with density (4) arises solely due to sputtering, we intentionally simplified the problem. It was noted earlier that the central region of the outer plate can be heated to a high temperature. The basis for neglecting its evaporation is the assumption that it is made of a refractory metal.

Strictly speaking, quantity (4) has a complex spatial distribution. First, even with a uniform current density distribution in the sputtering area, the spatial distribution of quantity (4) obeys the cosine law. Second, in a balanced magnetron with a three-layer target, the current density distribution *j*(*r*) is far from uniform. This is due to the magnetic field configuration. The first feature can be ignored, assuming that the substrate is sufficiently distant from the target plane. Furthermore, the substrate can be chosen to be of such a small area that the spatial non-uniformity of the sputtering material flux can be ignored. Regarding the non-uniformity of the current density distribution, we will discuss this feature when deriving the system of equations describing the target.

To deposit a film of a three-component equiatomic alloy, it is necessary to ensure the equality of the metal fluxes $Q_{i}{\left(r,\;\varphi \right)}_{z=0}=s_{i}J_{i}{\left(r,\;\varphi \right)}_{z=0}=a_{i}s_{i}j\left(r,\;\varphi \right)/e,\;i=1,\;2,\;3$ that generate the plates at *z* = 0:

(6) $$Q_{1}{\left(r,\;\varphi \right)}_{z=0}=Q_{2}{\left(r,\;\varphi \right)}_{z=0}=Q_{3}{\left(r,\;\varphi \right)}_{z=0}.$$

The condition expressed by the system of Equation (6) makes it possible to determine the angles α_2_ and α_3_. Due to the axial symmetry of the magnetic field in a balanced magnetron, the current density at the target $j\left(r,\;\varphi \right)$ loses its dependence on the angle. It follows that the quantities $Q_{i}{\left(r,\;\varphi \right)}_{z=0}\;\mathrm{and}\;J_{i}{\left(r,\;\varphi \right)}_{z=0},\;i=1,\;2,\;3$ are also independent of this angle. Taking this into account, we express system (6) in a more convenient relative form:

(7) $$\frac{Q_{1}{\left(r\right)}_{z=0}}{\sum Q_{i}{\left(r\right)}_{z=0}}=\frac{Q_{2}{\left(r\right)}_{z=0}}{\sum Q_{i}{\left(r\right)}_{z=0}}=\frac{Q_{3}{\left(r\right)}_{z=0}}{\sum Q_{i}{\left(r\right)}_{z=0}}=0.333.$$

Since (7) contains only two unknowns α_2_ and α_3_, it is sufficient to leave two equations in it, writing them, for example, in the form $3Q_{i}{\left(r\right)}_{z=0}=\sum Q_{i}{\left(r\right)}_{z=0},\;i=2,\;3$:

(8) $$\begin{array}{l} -Q_{3}{\left(r\right)}_{z=0}+2Q_{2}{\left(r\right)}_{z=0}-Q_{1}{\left(r\right)}_{z=0}=0,\\ 2Q_{3}{\left(r\right)}_{z=0}-Q_{2}{\left(r\right)}_{z=0}-Q_{1}{\left(r\right)}_{z=0}=0. \end{array}$$

If we take into account the dependence of metal fluxes on the discharge current density $Q_{i}{\left(r\right)}_{z=0}=s_{i}J_{i}{\left(r\right)}_{z=0}=a_{i}s_{i}j\left(r\right)/e,\;i=1,\;2,\;3$, then system (8) can be written as

(9) $$\begin{array}{l} -a_{3}s_{3}j\left(r\right)/e+2a_{2}s_{2}j\left(r\right)/e-a_{1}s_{1}j\left(r\right)/e=0,\\ 2a_{3}s_{3}j\left(r\right)/e-a_{2}s_{2}j\left(r\right)/e-a_{1}s_{1}j\left(r\right)/e=0. \end{array}$$

Ultimately, after obvious simplification, the system of Equation (9) takes the form

(10) $$\begin{array}{l} -a_{3}s_{3}+2a_{2}s_{2}-a_{1}s_{1}=0,\\ 2a_{3}s_{3}-a_{2}s_{2}-a_{1}s_{1}=0. \end{array}$$

The current density distribution is absent from the system of Equation (10). This fact leads to a very important conclusion that the result of calculating the geometric dimensions of the plates in a target with slots in the form of ring sectors with a given angle (see Figure 6) does not depend on the adopted distribution *j*(*r*).

Now we substitute into (10) the expressions for the sputtering zones on the plates obtained from (3):

(11) $$s_{1}=\frac{\alpha_{2}s}{180},\;s_{2}=\frac{\left(\alpha_{3}-\alpha_{2}\right)s}{180},\;s_{3}=s\left(1-\frac{\alpha_{3}}{180}\right).$$

Taking into account expressions (11), the system of Equation (10) takes its final form

(12) $$\begin{array}{l} -a_{3}\left(1-\frac{\alpha_{3}}{180}\right)+\frac{a_{2}}{90}\left(\alpha_{3}-\alpha_{2}\right)-\frac{a_{1}\alpha_{2}}{180}=0,\\ 2a_{3}\left(1-\frac{\alpha_{3}}{180}\right)-\frac{a_{2}}{180}\left(\alpha_{3}-\alpha_{2}\right)-\frac{a_{1}\alpha_{2}}{180}=0. \end{array}$$

The solution of system (12) in the form of values of angles α_2_ and α_3_ is more than obvious:

(13) $$\begin{array}{l} \alpha_{2}=\frac{180a_{2}a_{3}}{a_{1}a_{2}+a_{1}a_{3}+a_{2}a_{3}},\\ \alpha_{3}=\frac{180\left(a_{1}a_{3}+a_{2}a_{3}\right)}{a_{1}a_{2}+a_{1}a_{3}+a_{2}a_{3}}. \end{array}$$

The parameters of the problem, as follows from (13) and (4), are the sputtering and ion-electron emission coefficients of the metals that make up the three-layer target. Moreover, the solution to (13) is independent of the sizes of the target’s sputtering area *R*_01_ and *R*_02_.

### 2.3. TiTaNb Alloy Film

As already noted, films of three-component alloys based on the Ti-Ta system have higher quality. Along with those mentioned earlier, films of TiTaV [33], TiTaZr [34] and other alloys are being studied. Much attention has been paid to films of the medium-entropy TiTaNb alloy [35], so we will illustrate the use of a magnetron with the proposed three-layer target for their synthesis. As follows from the film composition, the target should contain titanium, tantalum, and niobium plates. The arrangement of the plates does not play a significant role. However, in the most general case, it is desirable to use the inner plate made of the metal with the lowest melting point. Placing this plate in either of the two positions is undesirable. Typically, there is no gap between the plates. Due to imperfect thermal contact, the middle and outer plates will be poorly cooled and, at a discharge current of several amperes, can be locally brought to the melting temperature. For this reason, we choose titanium as the material for the inner plate, tantalum for the middle plate, and niobium for the outer plate. For convenience, we use metal symbols as subscripts for the angles of the sectors cut into the plates: α_2_ ≡ α_Ta_ and α_3_ ≡ α_Nb_. The values of the physical parameters of the plate materials are given in Table 1.

Calculations were performed for the target mounted on a balanced magnetron with a diameter of 13 cm, which we use in our experimental studies. The annular sputtering area of the target of this magnetron is limited by the inner and outer radii *R*_01_ = 1.9 cm and *R*_02_ = 3.9 cm. The result of applying Formula (13) for this target is recorded in the corresponding row of Table 1. We determine the fraction of the sputtering zone of each plate in the sputtering area of the target by the expression

(14) $$\delta_{i}=\frac{s_{i}}{s},\;i=\mathrm{Ti},\;\mathrm{Ta},\;\mathrm{Nb}.$$

**Figure 8:**
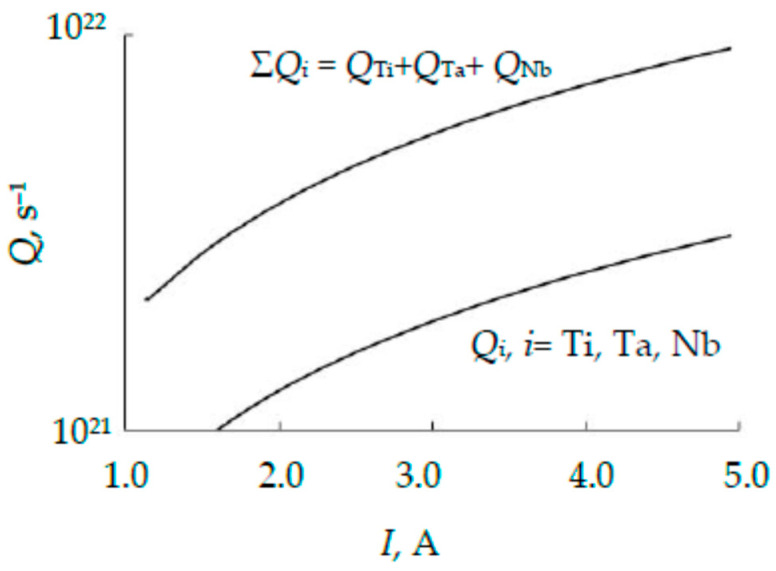
Dependences on the discharge current of the metal fluxes *Q_i_*, *i* = Ti, Ta, Nb and the total flow Σ*Q_i_*.

The results of calculating the sputtering zones on the wafers and their fractions using Formulas (3) and (14), respectively, are summarized in Table 1. Figure 8 shows the dependences of the metal fluxes ($Q_{i},\;i=\mathrm{Ti},\;\mathrm{Ta},\;\mathrm{Nb}$) generated by different wafers, calculated using Formula (4), on the discharge current, and the dependence of the total flux Σ*Q_i_*, which forms the alloy film. Figure 8 shows that the target generates three equal fluxes. This indicates that the composition of the TiTaNb alloy film deposited using the three-layer target under consideration may have a composition close to equiatomic.

Thus, the presented model makes it possible to design a three-layer target intended for the deposition of a film of a three-component equiatomic alloy. In conclusion, we note that the target type discussed here can be used to deposit films containing a larger number of components. To do this, simply increase the number of plates in the target and complicate the system of Equation (6) by adding new components.

The composition of films obtained under real experimental conditions may differ from the modeling results due to a number of errors. The most significant source (up to 10% [36]) is the assumption of the sputtering yield being independent of the ion energy in expression (4). In addition, the flux sputtered from the outer plate may be affected by heating and deformation of its central region. This drawback can be eliminated by simply changing the design of the plates. Instead of solid metal disks in which slots must be made, rings with an internal radius of *R*_01_ = 1.9 cm should be used. Finally, a minor error may be introduced by an error in the mechanical processing used to make the slots in the plates.

## 3. Four-Layer Target

### 3.1. Model for a Four-Layer Target

**Figure 9:**
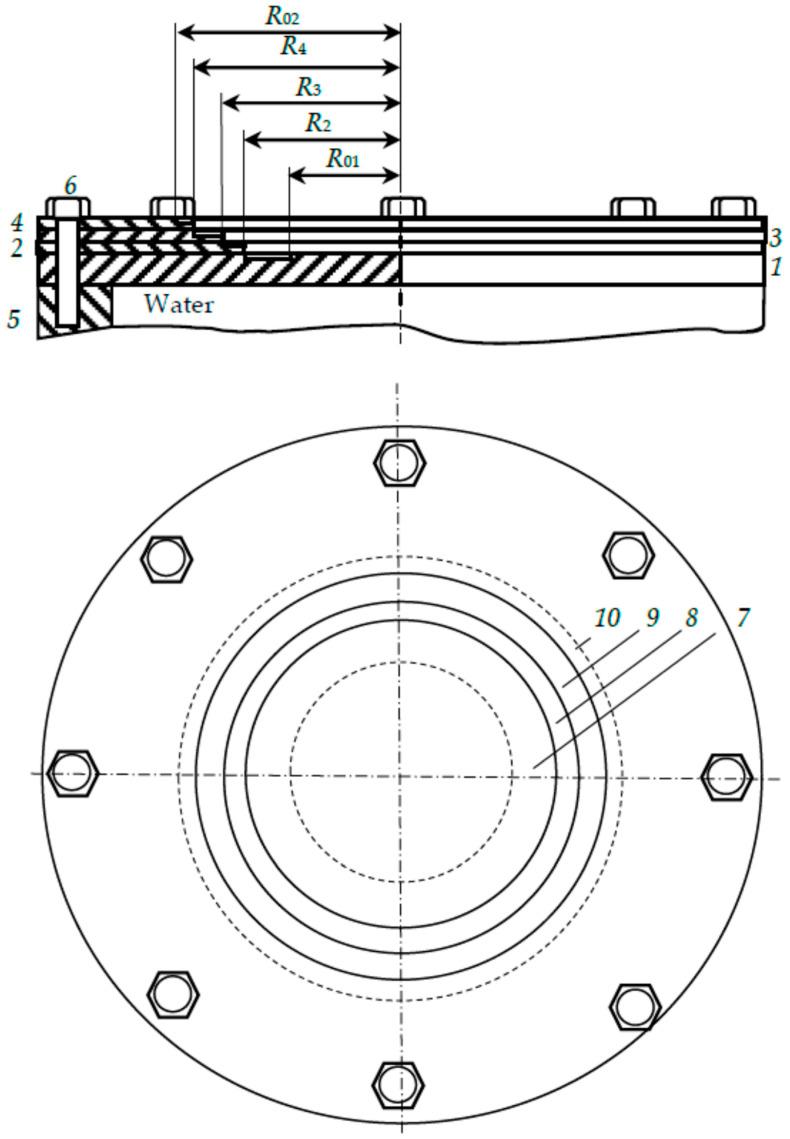
A schematic representation of a four-layer target containing the following plates: *1*—internal; *2* and *3*—first and second middle, respectively; *4*—external. In addition, the following are indicated; *5*—magnetron housing; *6*—mounting bolts; *7*–*10*—sputtering zones of the plates; *R*_01_ and *R*_02_—the inner and outer radius of the annular sputtering zone, respectively; *R*_2_, *R*_3_, *R*_4_—radii of the slots in the first, second middle and outer plates, respectively.

In this section, we will consider a model of a target designed for deposition of a film of a four-component alloy, which we will designate as M_1_M_2_M_3_M_4_. It will contain four plates, but of a different design. The plates in the new version of the multilayer target are ring-shaped. A schematic representation of this target is shown in Figure 9.

The use of plates of this shape significantly alters the thermal processes accompanying sputtering. Simulations for plates with the same slots as in Figure 3 and Figure 4 [28], as well as experimental studies, showed that maximum heating occurs in the central region of the plates, outside the sputtering area. Removing this area from the plates significantly changes the thermal process on the target. In the new design, heat dissipation from the sputtering area will occur only to the periphery, to the attachment points. This should significantly reduce the heating temperature and plate deformation.

The target in Figure 9 contains four sputtering metal plates *1*–*4*. We will assume that the target is designed for a balanced magnetron; therefore, as in the previous case, it has an annular sputtering area bounded by inner and outer radii *R*_01_ and *R*_02_, respectively. The plates are mounted on the same axis as the magnetron *5* and are rigidly attached to it with bolts *6*. The inner solid plate *1*, cooled by running water, the middle plates *2*, *3*, and the outer plate 4 are made of metals M_1_, M_2_, M_3_, and M_4_, respectively. Plates *2*–*4* are rings with inner radii *R*_2_, *R*_3_, and *R*_4_, respectively.

Plate *1* is a cooler, removing heat from the target. Plates *2*–*4* operate in free thermal mode, i.e., they are cooled by radiation and thermal conductivity.

The independent variables of the device are the discharge current, argon pressure, and the inner radii of the annular plates. To simplify further analysis, we will assume that the current density in the sputtering area bounded by radii *R*_01_ and *R*_02_, is uniformly distributed. This is a gross simplification of the problem. Its possible influence will be discussed below.

From Figure 9 it can be seen that the sputtered zones of plates *1*–*4* have the shape of rings with an area of:

(15) $$s_{1}=\pi \left(R_{2}^{2}-R_{01}^{2}\right);\;\;s_{2}=\pi \left(R_{3}^{2}-R_{2}^{2}\right);\;s_{3}=\pi \left(R_{4}^{2}-R_{3}^{2}\right);\;s_{4}=\pi \left(R_{02}^{2}-R_{4}^{2}\right).$$

As in the previous case, each plate, due to the argon ion flux, becomes a source of metal flux. Their densities are determined by expressions (4) and (5) for *i* = 1, 2, 3, 4.

The condition for the deposition of an equiatomic alloy (6), taking into account the assumption adopted for this case, is expressed in the form

(16) $$Q_{1}{\left(r\right)}_{z=0}=Q_{2}{\left(r\right)}_{z=0}=Q_{3}{\left(r\right)}_{z=0}=Q_{4}{\left(r\right)}_{z=0.}$$

Considering the axial symmetry of the magnetic field, let us write the system (16) by analogy with (7) in relative form:

(17) $$\frac{Q_{1}{\left(r\right)}_{z=0}}{\sum Q_{i}{\left(r\right)}_{z=0}}=\frac{Q_{2}{\left(r\right)}_{z=0}}{\sum Q_{i}{\left(r\right)}_{z=0}}=\frac{Q_{3}{\left(r\right)}_{z=0}}{\sum Q_{i}{\left(r\right)}_{z=0}}=\frac{Q_{4}{\left(r\right)}_{z=0}}{\sum Q_{i}{\left(r\right)}_{z=0}}=0.25.$$

Since system (17) contains only three unknowns *R*_2_, *R*_3_, and *R*_4_, it is sufficient to leave three equations in it, writing them, for example, in the form $4Q_{i}{\left(r\right)}_{z=0}=\sum Q_{i}{\left(r\right)}_{z=0},\;i=2,\;3,\;4$:

(18) $$\begin{array}{l} -Q_{4}{\left(r\right)}_{z=0}-Q_{3}{\left(r\right)}_{z=0}+3Q_{2}{\left(r\right)}_{z=0}-Q_{1}{\left(r\right)}_{z=0}=0,\\ -Q_{4}{\left(r\right)}_{z=0}+3Q_{3}{\left(r\right)}_{z=0}-Q_{2}{\left(r\right)}_{z=0}-Q_{1}{\left(r\right)}_{z=0}=0,\\ \;\;\;\;3Q_{4}{\left(r\right)}_{z=0}-Q_{3}{\left(r\right)}_{z=0}-Q_{2}{\left(r\right)}_{z=0}-Q_{1}{\left(r\right)}_{z=0}=0. \end{array}$$

(18) should take the form $Q_{i}{\left(r\right)}_{z=0}=s_{i}J_{i}{\left(r\right)}_{z=0}=a_{i}s_{i}j_{i}\left(r\right)/e,\;i=1,\;2,\;3,\;4$. If this expression is substituted into (18), we obtain a complex picture of the dependence of the equations in the system on the current density *j_i_*(*r*), *i* = 1, 2, 3, 4 in the sputtering zone of each plate. Such a problem can be practically solved only by a numerical method. However, as indicated earlier, we have simplified the problem. We assumed a uniform distribution of the current density $j_{i}\left(r\right)\approx j_{0}\;i=1,\;2,\;3,\;4$ in the entire sputtering area of the target, limited by radii *R*_01_ and *R*_02_. Here $j_{0}$ is the average current density in the sputtering area. This yields a flux in the form $Q_{i}\left(r\right)=Q_{i}\approx a_{i}s_{i}j_{0}/e,\;i=1,\;2,\;3,\;4$, which allows us to simplify system (18) by removing the dependence on the coordinate *r* from it. The simplification of the target model is done only to demonstrate the possibility of applying the condition of equality of fluxes (16) for a four-layer target in a simple analytical form.

We will perform further transformations similar to those we performed for the three-layer target. We will express the system of Equation (16) in a form suitable for numerical solution. Taking into account the flows in the form $Q_{i}=a_{i}s_{i}j_{0}/e,\;i=1,\;2,\;3,\;4$ and expressions (15), after simplification the system of Equation (18) takes the form:

(19) $$\begin{array}{l} -\left(R_{02}^{2}-R_{4}^{2}\right)a_{4}-\left(R_{4}^{2}-R_{3}^{2}\right)a_{3}+3\left(R_{3}^{2}-R_{2}^{2}\right)a_{2}-\left(R_{2}^{2}-R_{01}^{2}\right)a_{1}=0,\\ -\left(R_{02}^{2}-R_{4}^{2}\right)a_{4}+3\left(R_{4}^{2}-R_{3}^{2}\right)a_{3}-\left(R_{3}^{2}-R_{2}^{2}\right)a_{2}-\left(R_{2}^{2}-R_{01}^{2}\right)a_{1}=0,\\ \;\;3\left(R_{02}^{2}-R_{4}^{2}\right)a_{4}-\left(R_{4}^{2}-R_{3}^{2}\right)a_{3}-\left(R_{3}^{2}-R_{2}^{2}\right)a_{2}-\left(R_{2}^{2}-R_{01}^{2}\right)a_{1}=0. \end{array}$$

The solution to system (19) is the values of the radii of the ring-shaped plates *R*_2_, *R*_3_ and *R*_4_, which ensure the solution of the problem. The parameters of the problem, as follows from (5), are the sputtering yield and ion-electron emission coefficient of the metals that make up the four-layer target. In addition, the boundaries of the sputtering region must be known in the form of radii *R*_01_ and *R*_02_.

### 3.2. TiTaNbMo Alloy Film

Below, we will use as an example the proposed design of a target for the deposition of a film of the four-component TiTaNbMo alloy. This alloy is also classified as medium-entropic. Among many similar alloys, such as TiTaNbW [21], TiTaNbZr [22], TiZrNbMo [38,39,40], NbMoTaW [41,42], TaNbCrTi [43], TaNbHfZr [44], TiTaZrAg [45] and others, it is among the leaders in high-temperature corrosion resistance [46], and is therefore of interest as a structural material in the aerospace and automotive industries, chemical engineering, and other industries where structural elements are subject to heating in chemically active environments.

In the target intended for the deposition of alloy TiTaNbMo films, we will arrange the plates as follows: the inner one is Ti, the first middle one is Ta, the second middle one is Nb, and the outer one is Mo. As in the previous case, the order of the plates can be arbitrary. For convenience, we use metal symbols as subscripts for the plate radii: *R*_2_ ≡ *R*_Ta_, *R*_3_ ≡ *R*_Nb_, *R*_4_ ≡ *R*_Mo_. The values of the physical parameters of the plate materials are given in Table 2.

The solution of system (19) can be written in a general form that is easy to interpret. Assuming that the values of *R*_01_ and *R*_02_ are unknown, from (19) we obtain:

(20) $$\begin{array}{l} R_{\mathrm{Ta}}^{2}=0.702R_{01}^{2}+0.298R_{02}^{2},\\ R_{\mathrm{Nb}}^{2}=0.475R_{01}^{2}+0.525R_{02}^{2},\\ R_{\mathrm{Mo}}^{2}=0.176R_{01}^{2}+0.824R_{02}^{2}. \end{array}$$

From (20), it follows that the inner radii of the target’s annular plates are uniquely related to the parameters of the sputtering area of the target, which also has an annular shape. They are equal to the hypotenuses of right triangles, the legs of which are proportional to the inner and outer radii of this region.

We use solution (20) to analyze the particular case of the magnetron used in our experiments. The design of this device’s magnetic system provides an annular sputtering area with inner and outer radii of 1.9 and 3.9 cm, respectively. The area of this region is $\pi \left(R_{02}^{2}-R_{01}^{2}\right)\approx 36.4\;{\mathrm{cm}}^{2}$.

The values of the target plate radii calculated using Formula (20), and the corresponding areas of the sputtering regions (15) are shown in the bottom two rows of Table 2.

**Figure 10:**
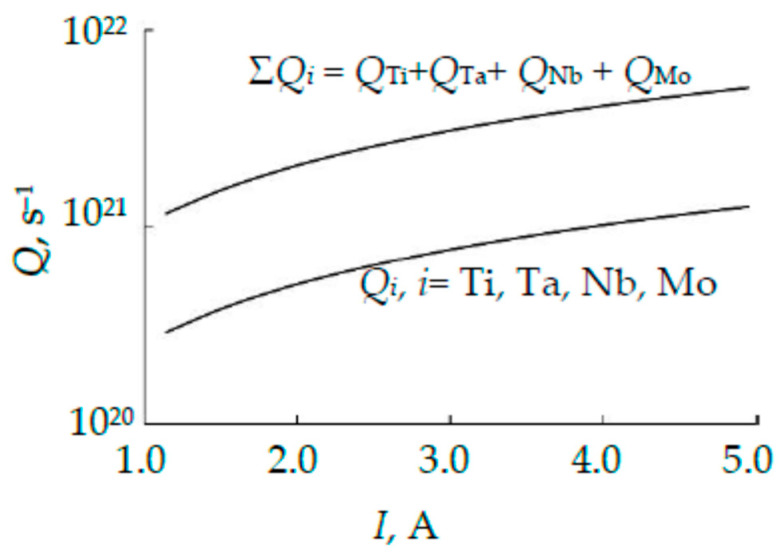
Dependences on the discharge current of the metal fluxes *Q*_i_, *i* = Ti, Ta, Nb, Mo and the total flux Σ*Q_i_*.

Figure 10 shows the dependences of the fluxes $Q_{i}=s_{i}J_{i},\;i=\mathrm{Ti},\;\mathrm{Ta},\;\mathrm{Nb},\;\mathrm{Mo}$ generated by different plates, calculated using Formula (4), and the total flux Σ*Q_i_* on the discharge current. Figure 10 shows that the target generates four equal fluxes. This gives hope that the TiTaNbMo alloy film deposited using a magnetron with the proposed four-layer target may have a composition close to equiatomic.

It is appropriate here to analyze the influence of the discharge current density distribution on the calculation results. We will perform this using the example of a simple two-layer target intended for the deposition of an equiatomic TiTa alloy. When removing the two outer plates from the problem for a four-component alloy, the right-hand side in (17) will be equal to 0.5. The only unknown in the resulting system is the inner radius *R*_2_ of the outer tantalum plate. System (17), subject to $j_{i}\left(r\right)\approx j_{0},\;i=1,\;2$, will be reduced to a single equation with one unknown *R*_2_, which, by analogy with (19), can be written as

(21) $$\left(R_{02}^{2}-R_{2}^{2}\right)a_{2}-\left(R_{2}^{2}-R_{01}^{2}\right)a_{1}=0.$$

For the plate parameters from Table 2, the solution to Equation (21) yields *R*_2_ = 2.94. This value defines the areas of the sputtering zones of both plates. For the inner titanium plate, this zone has the shape of a ring with an inner *R*_01_ = 1.9 cm and an outer radius of *R*_2_ = 2.94 cm, respectively. Its area, in accordance with (15), is *s*_1_ = 15.8 cm^2^. Similar values for the outer tantalum plate are *R*_2_ = 2.94 cm, *R*_02_ = 3.9 cm, and *s*_2_ = 20.6 cm^2^. But, if we take into account the uneven distribution of current, then the values $Q_{i}\left(r\right)=s_{i}J_{i}\left(r\right)=a_{i}s_{i}j_{i}\left(r\right)/e,\;i=1,\;2$ should be substituted into Equation (17). This ultimately, taking into account (15), leads to the equation

(22) $$\left(R_{02}^{2}-R_{2}^{2}\right)a_{2}j_{2}\left(r\right)-\left(R_{2}^{2}-R_{01}^{2}\right)a_{1}j_{1}\left(r\right)=0.$$

Compared to (21), Equation (22) cannot be solved analytically, since the current density and the area of the sputtering zones in (22) are interrelated. To estimate the error associated with this assumption, we assume that the sputtering process occurs at a discharge current of I, A. We simplify the problem by assuming uniform current distribution within the sputtering region of each plate. The value of the corresponding current is proportional to the area of the sputtering zone of the plate: $I_{i}\left(r\right)\approx I_{i0}=Is_{i}/\left(s_{1}+s_{2}\right),\;i=1,\;2$. Taking this into account, we determine the metal fluxes generated by both plates, writing them in the form

(23) $$Q_{i}=a_{i}I_{i}/e=a_{i}s_{i}I/\left[e\left(s_{1}+s_{2}\right)\right],\;i=1,\;2.$$

**Figure 11:**
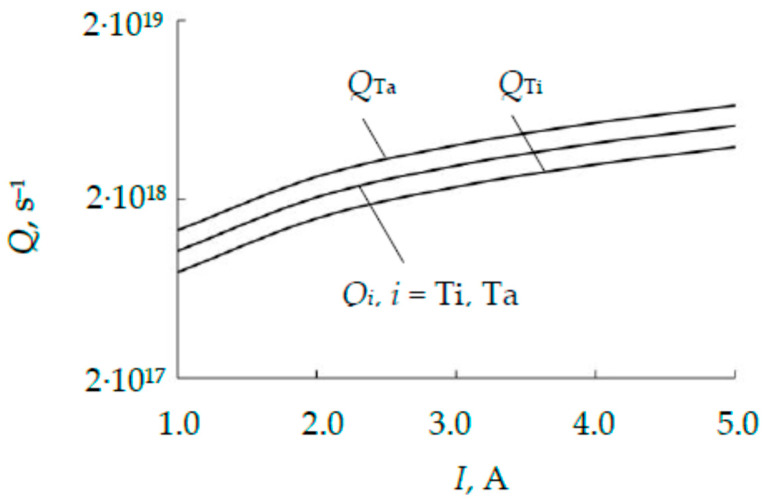
Dependences on the discharge current of the metal flows *Q*_Ta_ and *Q*_Ti_, calculated using (23) with the value *R*_2_ = 2.94 cm and the flows *Q_i_*, *i* = Ti, Ta, calculated using the iterative procedure.

Figure 11 shows the dependences of the flows of titanium and tantalum atoms on the discharge current, calculated using (23) with the value *R*_2_ = 2.94 cm, which is calculated using Equation (21).

From Figure 11, it is evident that the assumption of uniformity of the discharge current distribution in the target sputtering area leads to a difference in the tantalum and titanium fluxes of approximately 40%. As noted earlier, the calculation of the *R*_2_ value, which ensures equality of the fluxes, can be calculated using an iterative procedure in which *R*_2_ = 2.94 cm is taken as the initial value. Obviously, to solve the problem, it is necessary to reduce the flux of tantalum atoms. This can be achieved by increasing the value of *R*_2_. In such a simple case, it turned out to be sufficient to perform a selection without complex procedures. It turned out that equality *Q*_Ta_ = *Q*_Ti_ is achieved at *R*_2_ = 3.19 cm. These dependencies are designated in Figure 11 as *Q_i_*, *i* = Ti, Ta.

Thus, the methodology proposed in this subsection allows for an approximate analytical solution to the problem. This can be used as an initial approximation when refining the geometric dimensions of the annular plates using an iterative procedure.

## 4. Conclusions

In this communication, we show that equiatomic alloy thin films can be produced using a magnetron fitted with a novel “multilayer target” composed of multiple coaxial metal plates. Outer plates are slotted to expose underlying plates to sputtering, and we develop an analytical method to determine plate geometry by enforcing equality of sputtering fluxes. That condition reduces to a system of algebraic equations solved assuming a uniform discharge current density in the sputtering region. Two slot geometries are analyzed: sector-shaped slots (ring sectors), for which the geometry solution is independent of the current-density distribution, and ring-shaped plates with a removed central region, for which the method provides an approximate analytical solution. The multilayer-target paradigm therefore offers a practical route to controlled equiatomic deposition, with exact and approximate design solutions depending on slot geometry.

As examples, we present a calculation of the geometric dimensions of targets for a balanced magnetron system intended for the deposition of films of equiatomic alloys Ti_0.33_Ta_0.33_Nb_0.33_ and Ti_0.25_Ta_0.25_Nb_0.25_Mo_0.25_. This preliminary communication outlines a new multilayer-target paradigm for magnetron sputtering that—while supported by individual experimental results—has not yet directly confirmed equiatomic alloy film production; ongoing experimental verification is under way. The approach is grounded in established physical concepts of metal sputtering in argon and enables fabrication of nanostructured medium- and high-entropy alloy films with specified composition, aligning with the Special Issue’s focus on functional nanomaterials. The proposed target designs are scalable beyond three or four wafers and may accommodate many plates, making them suitable for deposition of high-entropy alloys. Furthermore, multilayer targets are applicable for reactive deposition of films of solid solutions of simple compounds (e.g., oxides and nitrides). In such cases, the target is sputtered in a gas mixture containing argon and a reactive gas. The task of depositing a film of a given chemical composition is significantly complicated by the introduction of an additional independent variable: the input flow of the reactive gas.

## Figures and Tables

**Table 1:** Physical parameters of plate materials and calculation results for a three-layer target.

Parameter	*i*		
	Ti	Ta	Nb
*S_i_* [36]	0.32	0.4	0.3
*γ_i_* [37]	0.1100	0.0577	0.0444
*a_i_*	0.288	0.378	0.287
α*_i_*, grad	-	65	115
*s_i_*, cm^2^	13.2	10.1	13.1
δ*_i_*	0.363	0.276	0.361

**Table 2:** Parameters of plate materials and calculation results for a four-layer target.

Parameter	Metal			
	Ti	Ta	Nb	Mo
*S_i_* [36]	0.32	0.4	0.3	0.5
*γ_i_* [37]	0.1100	0.0577	0.0444	0.0222
*a_i_*	0.288	0.378	0.287	0.489
*R_i_*, cm	-	2.7	3.1	3.6
*s_i_*, cm^2^	10.9	8.2	11.0	6.4

## Data Availability

The original contributions presented in this study are included in the article. Further inquiries can be directed to the corresponding author.
